# Development of Functionalized Poly(ε-caprolactone)/Hydroxyapatite Scaffolds via Electrospinning 3D for Enhanced Bone Regeneration

**DOI:** 10.1021/acsomega.4c05264

**Published:** 2024-10-30

**Authors:** Maria José da Silva Lima, Etelino Feijó de Melo, Kleber G. B. Alves, Fabrício Bezerra de Sá, Severino Alves Júnior

**Affiliations:** † Departamento de Química Fundamental, Universidade Federal de Pernambuco, Recife 50670-901, Pernambuco, Brazil; ‡ Instituto Federal de Educação, Ciência e Tecnologia de Pernambuco, Vitória de Santo Antão 55600-000, Pernambuco, Brazil; § Departamento de Engenharia Mecânica, Universidade Federal de Pernambuco, Recife 50670-901, Pernambuco, Brazil; ∥ Departamento de Morfologia e Fisiologia Animal, Universidade Federal Rural de Pernambuco, Recife 52171-900, Pernambuco, Brazil

## Abstract

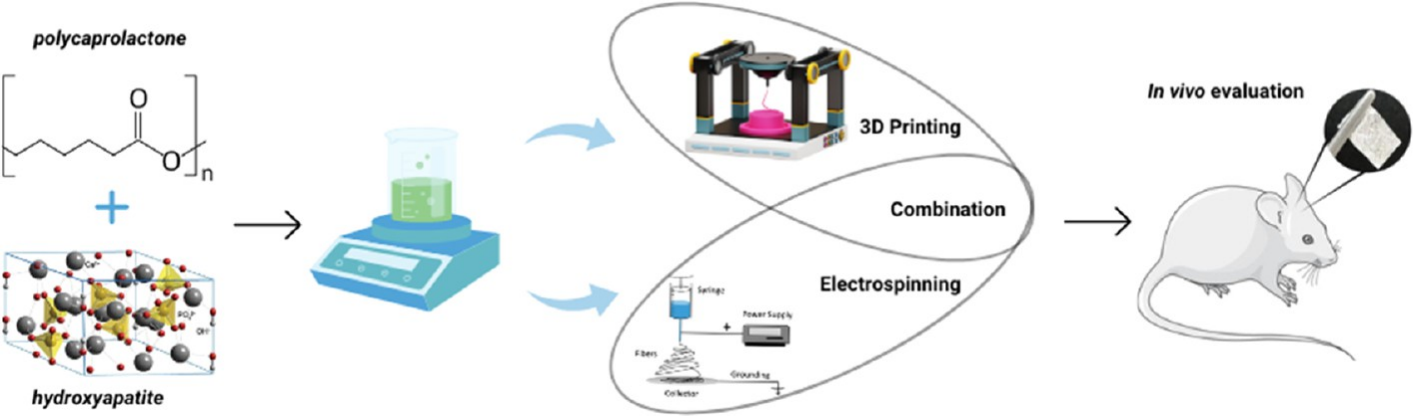

Functionalized scaffolds based on biodegradable polymers are materials used in bone tissue engineering. This study presents the development of functionalized fibrous scaffolds, fabricated from poly(ε-caprolactone) (PCL) and hydroxyapatite (HA). To produce this material, a short-distance electrospinning (ES) system was developed by adapting a 3D printer. The morphology and chemical properties of the scaffolds were evaluated using scanning electron microscopy, X-ray diffraction, Fourier-transform infrared spectroscopy, and thermogravimetric analysis. The results confirmed the porous structure and the presence of hydroxyapatite throughout the entire scaffold area. Mechanical tests indicated good elasticity and tensile strength of the scaffolds, favorable for bone regeneration. In vitro tests showed high levels of cell viability. Furthermore, in vivo experiments using a calvarial defect model in rats demonstrated that the PCL/HA scaffold promoted enhanced bone regeneration. Therefore, the PCL/HA scaffold developed through the adapted electrospinning system shows promise for bone repair.

## 1 Introduction

Bone replacement procedures or grafts are widely used in the treatment of tissues damaged by severe diseases, trauma, or aging-related factors. This treatment can be performed on various parts of the body, ranging from dental structures to craniofacial areas, thereby demonstrating broad applicability.^1,2^ Despite its importance, tissue transplantation procedures still present many limitations, ranging from economic infeasibility for many patients to issues directly related to the mechanism type. For instance, grafts can cause donor site morbidity, disease transmission risks, and even rejection, depending on the method.^1,3^

In this context, tissue engineering has gained prominence as it aims to regenerate or repair the functions of diseased tissues that do not heal spontaneously, such as bone, emerging as a promising field to overcome the challenges of conventional graft treatments.^4^ The architecture of materials designed for regeneration must facilitate cell adhesion, proliferation, and differentiation,^3,5^ while also meeting the mechanical requirements necessary for effective tissue regeneration.^6,7^ Thus, tissue engineering is based on three essential elements: cells, growth factors, and scaffolds.^1,2^

Scaffolds are three-dimensional structures^6^ that can be produced from nano and microfibers, hence referred to as fibrous, playing an essential role in the success of tissue engineering processes. They interact with cells and growth factors to regenerate or repair specific tissues, providing good support for cellular accommodation and guiding their growth.^1,5^ A scaffold must possess a porous structure with interconnected pore networks of appropriate sizes to ensure the supply of necessary nutrients for cell migration and oxygenation.^8,9^ The alignment of these fibers can enhance the material’s mechanical and biological properties, as well as promote better osteogenic differentiation. These requirements make the design process of this material quite challenging and complex, especially when traditional manufacturing techniques are used.^5^ Currently, some techniques have shown promise in overcoming these limitations by introducing relatively new approaches to the fabrication of these systems, including methods such as additive manufacturing and electrospinning.^9^

Electrospinning (ES) is a simple technique in which aligned or random micro and nanofibers can be prepared for various applications. This method requires a polymer solution of appropriate viscosity, which is transferred to a syringe.^10^ An electric field is then applied between the syringe tip and a metallic collector positioned at a predefined distance.^11^ Due to the electric potential difference between the collector and the syringe tip, micro and nanofibers are spun and deposited onto the metallic collector. Finally, the solvent evaporates, forming a mat of dry fibers on the collector. Although this technique demonstrates significant potential for scaffold preparation and yields fibers with desirable characteristics for cell attachment, it is constrained by limitations in the fabrication of scaffolds with well-defined structures. This limitation consequently diminishes the capacity to control specific properties tailored for particular applications, such as bone tissue engineering.^12−16^

Additive manufacturing, in turn, is a family of techniques that produce a 3D object through the layer-by-layer addition of a material, which can be polymeric, metallic, or ceramic, in the form of powder, filament, curable resin, or other formats. The use of additive manufacturing techniques to fabricate scaffolds allows researchers to create complex geometries with controlled porosities, capable of producing microinterconnections that promote appropriate cellular behavior.^11,17,18^

Previous studies have demonstrated that the preparation of appropriate scaffolds using a hybrid material composition and combining 3D printing and ES techniques to form hierarchical structures with suitable characteristics can be effective in bone regeneration.^19−23^ Researchers assert that this combination of techniques results in a multistructural scaffold capable of meeting the ideal criteria for bone defect repair.^24^

Therefore, the combination of 3D printing with the ES method appears to be highly advantageous. 3D printing can provide a predesigned porous structure, while the electrospinning technique can coat the scaffold surfaces with fibers. However, due to the limitation of the distance between the printer base and the substrate nozzle, it is necessary to adjust the ES technique, known as near-field electrospinning.^25^

Near-field ES can produce patterned fibers using a significantly shorter distance and reduced voltage compared to the conventional method.^26,27^ Reducing the distance between the nozzle tip and the collector allows for better control in the straight-line deposition of fibers. When the distance is properly adjusted, near-field ES minimizes bending instabilities and enables the separation of electrospun fibers.^28^ This technique has already enabled the production of fibers from various substances, such as polystyrene (PS),^28^ PCL,^29,30^ and polyvinylpyrrolidone (PVP),^30,31^ with precise control during deposition. Besides the technique used, the biomaterials employed in the preparation of scaffolds play a crucial role in achieving optimal results.

Among these biomaterials, hydroxyapatite (HA) stands out as the primary component of the bone matrix. Bone is a natural ceramic composite composed of collagen fibrils that contain embedded nanocrystalline inorganic materials and rods. Thus, synthetic HA has broad applicability in bone regeneration due to its biological characteristics.^8,9^ The close resemblance of synthetic HA to natural bone has led to extensive research efforts to use it as a component of bone substitutes. Nanoscale HA particles have proven to be an osteoconductive material.^6^ Another noteworthy biomaterial is poly(ε-caprolactone) (PCL), a biodegradable polymer with remarkable biocompatibility and high mechanical strength.^11^ PCL is frequently used as a polymer matrix in composites, including osteogenic inorganic phases that promote new blood vessel formation and stimulate bone cell formation, and osteoinductive phases that induce stem cells to differentiate into osteogenic cells upon contact with the bone matrix.^6^ In this context, this study demonstrates the development of a near-field ES system adapted to a 3D printer for the fabrication of scaffolds with oriented fiber deposition, which could pave the way for structured printing of tissues.

## 2 Materials and Methods

### 2.1 Materials

Materials including polycaprolactone polymer (PCL, *M*_w_ = 8000), calcium sulfate hemihydrate (CaSO_4_·**1**/**2**H_2_O), ammonium hydroxide (NH_4_OH), and monobasic ammonium phosphate (NH_4_H_2_PO_4_) were purchased from Sigma-Aldrich (St Louis, Missouri). Dichloromethane (CH_2_Cl_2_) was acquired from Neon Commercial (Suzano, SP, Brazil). Cell culture reagents, such as RPMI 1640 culture medium supplemented with 10% (w/v) fetal bovine serum, were obtained from Sigma-Aldrich (St. Louis, Missouri). Vero cells were sourced from the Rare Earth Laboratory of the Department of Fundamental Chemistry at the Federal University of Pernambuco (BSTR, dQF/UFPE, Brazil). For in vivo experiments, all animals underwent an identical therapeutic protocol subsequently elucidated.

### 2.2 3D Printer Adaptation

**Figure 1:**
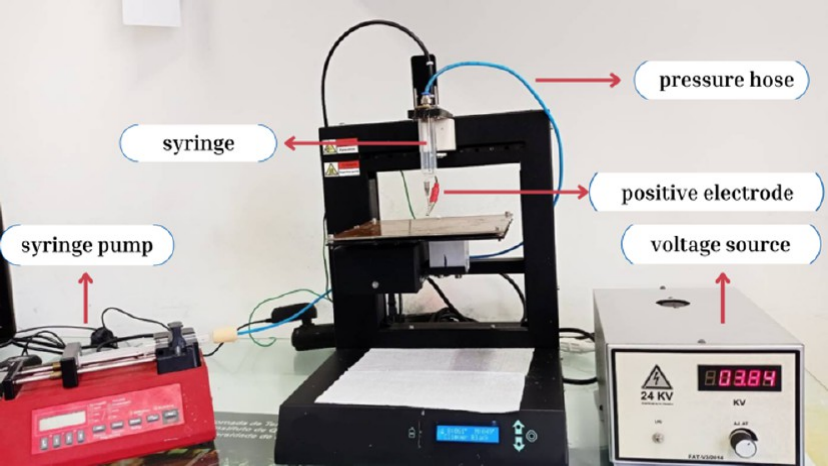
Electrospinning system after adaptation in a 3D printer and its main components.

Initially, a Cliever 3D printer (CL-1 Black Edition) was employed to create the necessary adaptations (see Figure 1). The printer settings used were as initially specified by the manufacturer.^32^ The heating base and the printing nozzle, responsible for heating and extruding the filament, were first removed to enable the required modifications. These components were equipped with NTC (Negative Temperature Coefficient) thermistors responsible for transmitting temperature signals from the respective heating elements to the controller board (equipment CPU). To facilitate printer operation without heating, the NTC thermistors were replaced with fixed resistors that restrict the flow of electrical current. This adjustment permitted the initiation of the ES process at room temperature, which was determined as suitable for commencing electrospun fiber production. The heating base was substituted with a virgin fiberglass printed circuit board of matching dimensions. This circuit board was connected to the negative terminal of the high-voltage source commonly utilized in conventional ES systems. After removing the printing nozzle, a support structure was fabricated to accommodate the syringe, a pressure hose, and the positive electrode connected to the high-voltage source. The pneumatic pressure hose, with a diameter of 4 mm, played a crucial role in supplying air pressure to the bronze piston, enabling the controlled flow of the polymeric suspension toward the needle tip. An infusion pump regulated this injection process, often called a syringe pump. The bronze pistons were meticulously designed to permit the passage of air pressure when coupled to the syringe.

For the production of scaffolds using the adapted ES system, a 3D model was developed based on a design created in specialized modeling software called Cliever Studio, version Windows 7+, where the scaffolds to be printed were designed in a rectangular shape. This model was subsequently used for printing in a meticulous process. During printing, the system was directed to deposit fibers both vertically and horizontally, following a predefined pattern. This method enabled the creation of predetermined pores in the final object.

### 2.3 Preparation of Hydroxyapatite (HA) Powder

HA was synthesized using a chemical precipitation method. In brief, 500 mL of a solution containing calcium sulfate hemihydrate (0.1 mol L^–1^) was placed under continuous mechanical agitation throughout the synthesis process. The initial pH of the solution was set at 6.0 and gradually raised to 10 by stepwise additions of aliquots of ammonium hydroxide solution (3 mol L^–1^). Subsequently, 500 mL of monobasic ammonium phosphate solution (0.06 mol L^–1^) was introduced into the system at a controlled incorporation rate of 20 mL per minute. During the synthesis, the pH was closely monitored and adjusted to a range of 10 by gradually adding ammonium hydroxide solution. After adding reagents, the system was continuously and vigorously stirred at 25 °C for 1 h to ensure uniform mixing. A white precipitate formed during this stage, which was then allowed to age for 2 days to facilitate hydroxyapatite formation. The synthesized hydroxyapatite was subsequently separated by filtration and washed with ultrapure water until the pH reached 7. It was then dried in an oven at 90 °C for 24 h to eliminate residual solvents. Finally, the material was calcined in hot air in an oven for 2 h at 900 °C.

### 2.4 PCL/HA Scaffold Electrospinning

The PCL was dissolved in dichloromethane, and HA was dispersed through an ultrasonic device in dichloromethane at room temperature and then mixed. The mixture was stirred for 4 h to prepare the ES suspension of PCL/hydroxyapatite (w/v = 8.4%). The mass ratio of PCL to HA (w/w) was 9:1. The electrospinning procedure was carried out as follows: the PCL/HA solution was loaded into a plastic syringe, and the syringes were set up on syringe pumps, operating at a rate of 0.5 mL per hour. Subsequently, the PCL/HA solution was electrospun using a voltage of 3.90 kV. Aluminum foil was used as a collector, with the distance between the collector and the steel needle tip adjusted to 10 mm. After ES, the PCL/HA nanofiber films were air-dried for 2 h.

### 2.5 Characterization of Synthetic Hydroxyapatite and Scaffold

#### 2.5.1 Morphology

The structures and surface morphology of the HA samples and scaffolds were examined using a scanning electron microscope (SEM, TESCAN-MIRA-3, England). Prior to SEM imaging, the dry samples were sputter-coated with gold for 40 s, and the acceleration voltage was set to 10 kV. The SEM images were analyzed using ImageJ software (USA). Specifically, the software was employed to measure the diameter of the electrospun nanofibers and the average diameter of the synthesized hydroxyapatite. The dispersion of the electrospun nanofibers was assessed by calculating the parameters based on a random selection of 200 nanofibers, in triplicate (*n* = 3).

#### 2.5.2 Physical and Chemical Characterization the Synthetic Hydroxyapatite

Synthetic hydroxyapatite was subjected to evaluation through Fourier transform infrared spectroscopy (FTIR), X-ray diffraction (XRD), and thermogravimetric analysis (TGA). Infrared measurements were specifically conducted using a BRUKER IFS66 FTIR spectrometer, while XRD analysis was performed employing a RIGAKU SMARTLAB X-ray diffractometer (Rigaku, Japan) using the Cu K (= 1.54) radiation. The TGA assessment of the synthesized hydroxyapatite was carried out using a thermal analyzer (SHIMADZU DTG-60H). During the experiment, synthetic air was employed as the carrier gas at a flow rate of 100 mL min^–1^, and the heating rate was maintained at 10 °C min^–1^ from room temperature up to 700 °C.

#### 2.5.3 Physical and Chemical Characterization of Electrospun Scaffolds

Electrospun scaffolds were assessed through FTIR and TGA. During the experiment, synthetic air served as the carrier gas, with a flow rate of 100 mL min^–1^, and the heating rate was maintained at 10 °C min^–1^, ranging from room temperature to 600 °C.

#### 2.5.4 Tensile Mechanical Properties of Electrospun Nanofibers

The tensile mechanical properties of all the test specimens, with the following dimensions: 16 × 20 × 0.5 mm^3^ (*n* = 5), were determined using a universal testing machine (EMIC DL 10 000) equipped with a Trd 22 cell in film tension mode. The matrices were secured to the flat surface of a stainless steel base plate. The tests were conducted at a force application rate of 5 N/min. The tensile strength was defined as the breaking stress endured by the sample during a tensile test.

#### 2.5.5 Swelling Rate Measurement

Three samples from each group were cut into square specimens measuring 2 × 2 cm^2^, with a controlled thickness of approximately 1 mm. These samples were immersed in distilled water at 37 °C. At each specified time point, the samples were removed, dried, reweighed, and the swelling rate was determined according to the eq 1
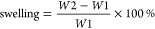
1Where W1 is the initial weight of the material, and W2 is the weight of the material after soaking in distilled water.

#### 2.5.6 MTT Assay

To evaluate the bioactivity of the scaffolds in promoting the proliferation of Vero cells (a model that mimics normal cells), an MTT assay was conducted. This assay quantified the production of purple formazan resulting from the mitochondrial reduction of blue tetrazolium bromide at various time points after cell seeding on the nanofibers. Sterilized samples were placed in a 12-well plate with a diameter of 35 mm, and the assay was performed in triplicate. Subsequently, 500 μL of α minimal nonosteogenic essential medium was added, and cells were seeded at a density of 106 cells per well. After a 3-day incubation period, the supernatant medium was carefully aspirated, and the scaffolds were washed twice with a PBS solution. The cell-seeded scaffolds were incubated with 500 μL of a 500 μg/mL MTT solution for 4 h at 37 °C, after which the supernatant solution was removed. The resulting purple formazan crystals were extracted by adding 250 μL of dimethyl sulfoxide (Sigma-Aldrich, St. Louis, Missouri) to each well for 10 min. Wells containing Vero cells without scaffolds served as the control. The absorbance of the extract was measured at 570 nm, concerning 690 nm for the medium, using a Symergy HT multisensing microplate reader (Symergy HT, BioTek, USA). The quantity of formazan produced was determined based on the microplate reader data, with values obtained from control wells subtracted from the measured values. Cell viability, correlated with optical density, was assessed by normalizing the values to those from the positive control wells.

#### 2.5.7 Animal Study for Bone Regeneration In Vivo

After receiving approval from the Animal Use Ethics Committee at the Federal Rural University of Pernambuco (UFRPE) under license number 4838071020 (ID 000511), 8-weekold male (*Rattus norvegicus* Wistar) rats, each weighing 250 g, were selected from the Morphology and Animal Physiology Department at UFRPE for this study (*n* = 8). To assess the regenerative potential of the scaffolds, a sagittal skin incision approximately 2.5 cm long was made using a No. 15 scalpel blade, creating circular defects that allowed for gentle tissue exposure to expose the parietal bone. A five-millimeter diameter defect (injury) was then carefully made in the parietal bone on the left side of the cranial vault of each rat, with the dura mater membrane serving as its boundary, using a trephine surgical drill coupled to a lowspeed motor. The rats were divided into two groups, each containing four individuals. One group served as the control group (CG), where animals did not receive any bone defect fillings, and the other group was the experimental group (EG), where scaffolds were used as grafts. Weekly clinical assessments were conducted for each group, and after euthanasia, histopathological evaluations were performed. A fragment of scaffold, approximately 6 mm in size, was inserted into the surgical defect and covered with sutured skin. Following the surgical procedures in each subgroup, the animals were placed in individual cages for 10 days and then reunited until the scheduled date for euthanasia. All animals received the same therapeutic protocol, including Benzathine Penicillin at a dose of 24 000 IU/kg administered intramuscularly as a single dose, Meloxicam at a dose of 1 mg/kg administered subcutaneously for 3 days, and Tramadol hydrochloride at a dose of 5 mg/kg administered subcutaneously for 3 days. Daily clinical monitoring was maintained to observe the progress of the healing processes and the animals’ general condition. Euthanasia was carried out through intraperitoneal injections of ketamine at a dose of 90 mg/kg combined with xylazine at a dose of 10 mg/kg, followed by intraperitoneal administration of sodium thiopental at a dose of 150 mg/kg. Subsequently, a perfusion was performed with a 0.9% physiological sodium chloride solution injected through the left ventricle and drained from the right atrium, followed by another perfusion with 10% formaldehyde. The heads of the animals were then removed, immersed in 10% formaldehyde for 72 h for fixation, and stored in 70% alcohol.

#### 2.5.8 Histological Evaluation

The skulls were defleshed, retaining only the parietal and frontal bones. Subsequently, they underwent a progressive alcohol-based dehydration process, followed by clearing in xylene, immersion in paraffin, and block preparation. Thin histological sections, measuring 5 μm in thickness, were obtained using a rotary microtome and stained with hematoxylin and eosin. Clinical monitoring was maintained to observe the progress of the healing processes and the animals’ general condition.

## 3 Results and Discussion

### 3.1 Morphological Characterization of HA Particles and PCL/HA Scaffolds

**Figure 2:**
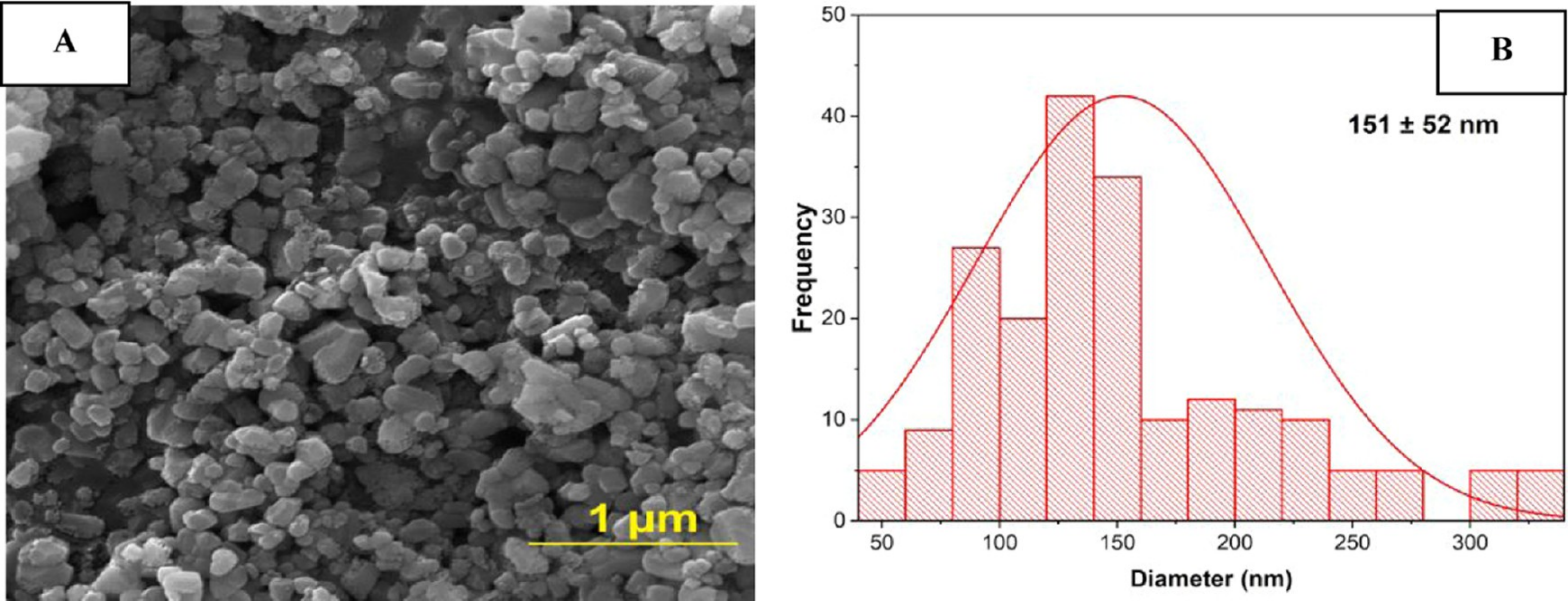
(A) SEM micrograph for the prepared HA (B) histogram of the mean particle size distribution for hydroxyapatite with standard deviation values (*n* = 3).

The morphological characteristic of the HA particles is showed in SEM images that revealed the presence of clusters of nanometer-sized particles characterized by a uniform distribution and samples reveal the presence of roughly spherical and round shapes, as depicted in Figure 2A. And Figure 2B displays a statistical analysis of the distribution and average size of these particles, generated using the open-source software ImageJ. Through statistical analysis, the average size of HA particles was determined to be 151 nm, with a standard deviation of ±52 nm for the analysis.

**Figure 3:**
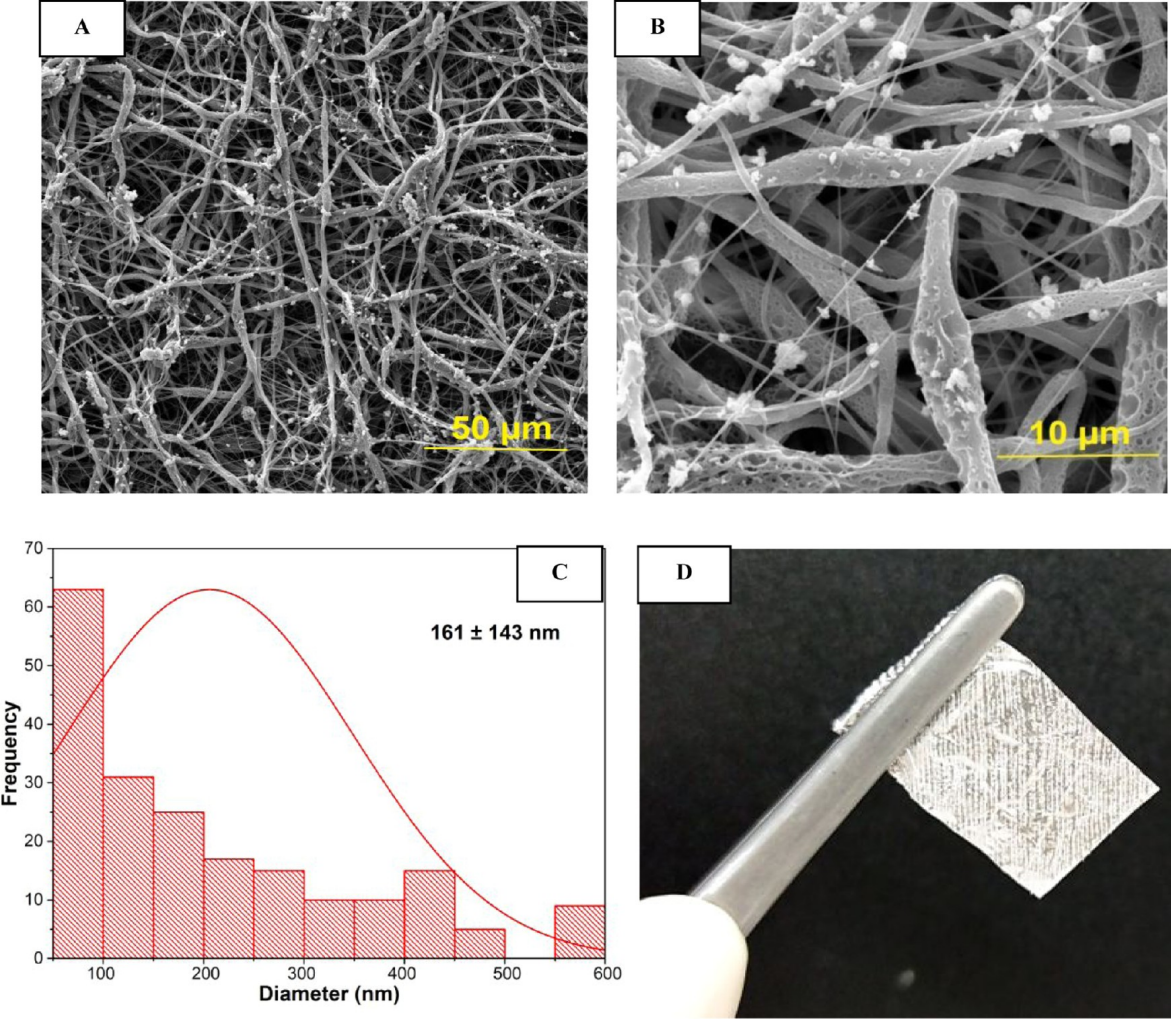
SEM images and fiber diameter distribution for PCL/HA scaffolds: (A) 10 000× magnification; (B) 15 000× magnification; (C) fiber diameter histogram with standard deviation values (*n* = 3); (D) Scaffold produced with oriented fiber writing.

The SEM images of the produced scaffolds displayed clusters of hydroxyapatite particles deposited on the fiber structure, visually confirming their integration according with Figure 3A,B. In this case, the fiber diameter ranged from 1 to 600 nm, with an average fiber diameter of 161 nm, Histogram on Figure 3C. Adapting ES technology to a 3D printer has enabled the production of electrospun scaffolds with oriented fiber. An illustration of the resulting material is presented in Figure 3D.

### 3.2 FTIR Characterization of Hydroxyapatite and PCL/HA Scaffolds

**Figure 4:**
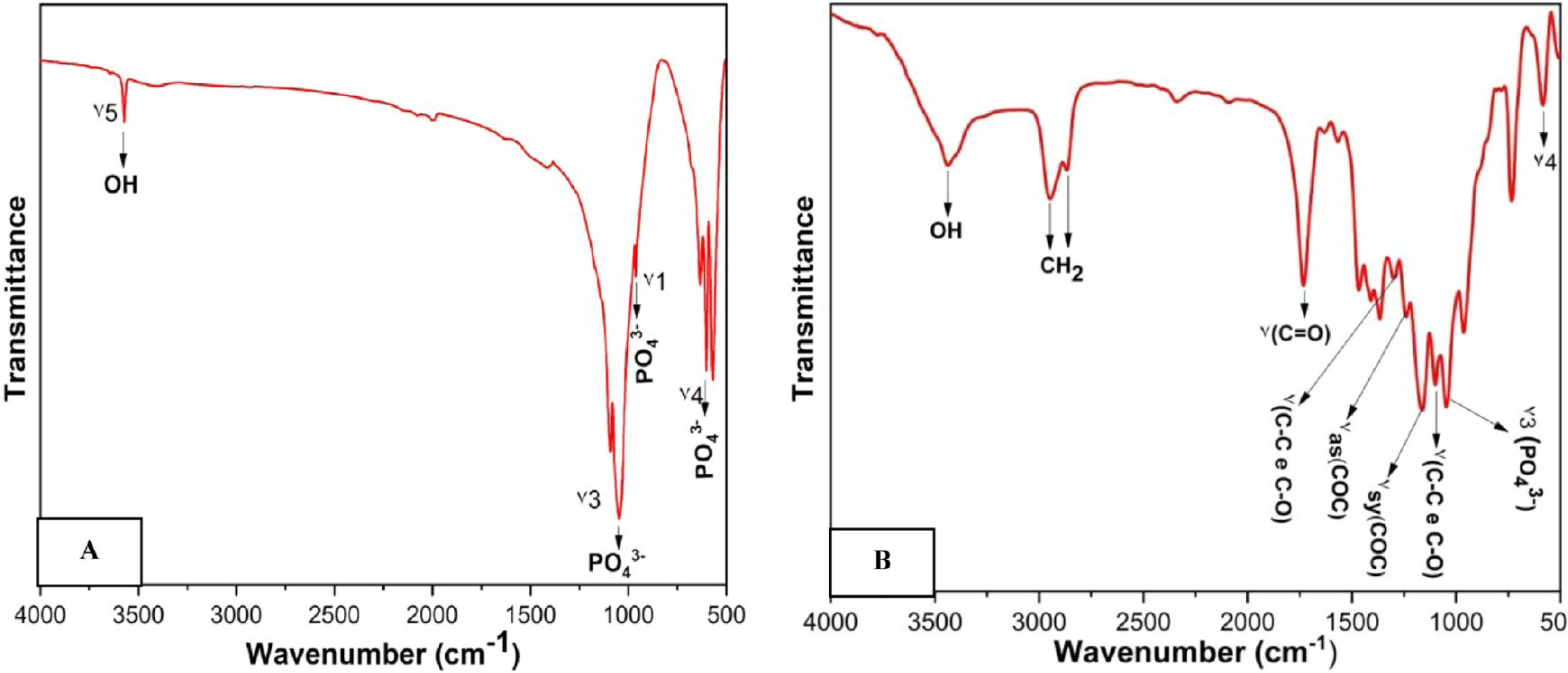
FTIR spectrum for (A) hydroxyapatite calcined at 900 °C and (B) PCL/HA Scaffolds.

The (HA) underwent initial characterization through FTIR spectroscopy to analyze the vibrational bands of its functional groups. Figure 4A illustrates the FTIR spectrum of the hydroxyapatite powder heat-treated at 900 °C. A prominent absorption band associated with HA appears at 3572 cm^–1^, attributed to the hydroxyl O–H stretching (vibration mode ν_5_). Furthermore, distinctive absorption bands are observed at 1047 cm^–1^ (ν_3_) and 962 cm^–1^ (ν_1_) for the asymmetric and symmetric stretching of the phosphate ion (PO_4_^3–^), respectively. The absorption bands exhibiting vibrations at 602 and 569 cm^–1^ correspond to the O–P–O strain in either the phosphate ion (PO_4_^3–^) or the hydrogen phosphate ion (HPO_4_^2–^), denoted as ν_4_.^33,34^

The FTIR spectra of PCL/HA scaffolds are presented in Figure 4B. In the scaffold spectrum, most observed bands correspond to both PCL and HA materials, confirming their presence. The bands associated with PCL include C=O, C–O, and C–H, while the bands attributed to HA are P–O and O–H. The FTIR spectrum for the PCL fiber exhibits features consistent with a linear aliphatic polyester in accordance with its structure below.
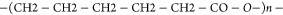
The bands observed at 2946 and 2868 cm^–1^ correspond to the asymmetric and symmetric axial deformation of the CH_2_ group, respectively. A strong-intensity band at 1729 cm^–1^ is attributed to the stretching vibration of the carbonyl (–C=O) bond. The band at 1295 cm^–1^ is assigned to the structural sites of C–C and C–O in the crystalline phase, while the band at 1107 cm^–1^ corresponds to the vibrations of C–O and C–C in the amorphous phase of the polymer. The characteristic band of the asymmetric C–O–C vibration is observed at 1249 cm^–1^, and the band at 1164 cm^–1^ corresponds to the symmetric O–C–O vibration.^35−37^ The vibrational mode of phosphate stretching (ν_3_ PO_4_^3–^) is observed at 1045 cm^–1^. The vibrational band (ν_4_) was detected at 583 cm^–1^. Furthermore, the band at 3450 cm^–1^ is associated with OH in HA. The results confirm the successful incorporation of hydroxyapatite particles into the fiber structure.

### 3.3 X-ray Diffraction Analysis of Hydroxyapatite

**Figure 5:**
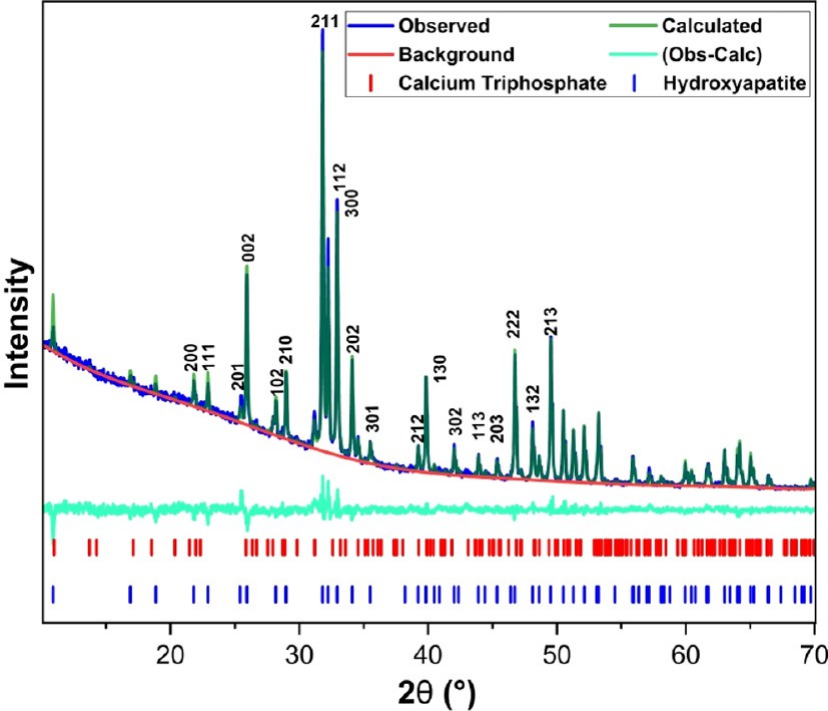
X-ray diffraction pattern of hydroxyapatite calcined at 900 °C.

The XRD analysis of HA powder treated at 900 °C is depicted in Figure 5.

The diffraction peaks were compared with reference cards from the ICSD database. The formation of HA with a hexagonal crystalline structure [Ca_10_(PO_4_)_6_(OH)_2_] with *P*63/*m* space group was confirmed, consistent with ICSD 16742 standards. As indicated in Figure 5, the main characteristic lines of hydroxyapatite (ICSD 16742) overlay well with the diffraction peaks of the synthesized material. Additionally, peaks representative of Tricalcium Phosphate (TCP), Ca_3_(PO_4_)_2_, with a rhombohedral crystal system and *R*3*c* space group (ICSD 6191), were also present at 2θ = 31.0°. The presence of TCP, which is also a biomaterial, can be justified by the thermal decomposition of HA during calcination.

**Figure 6:**
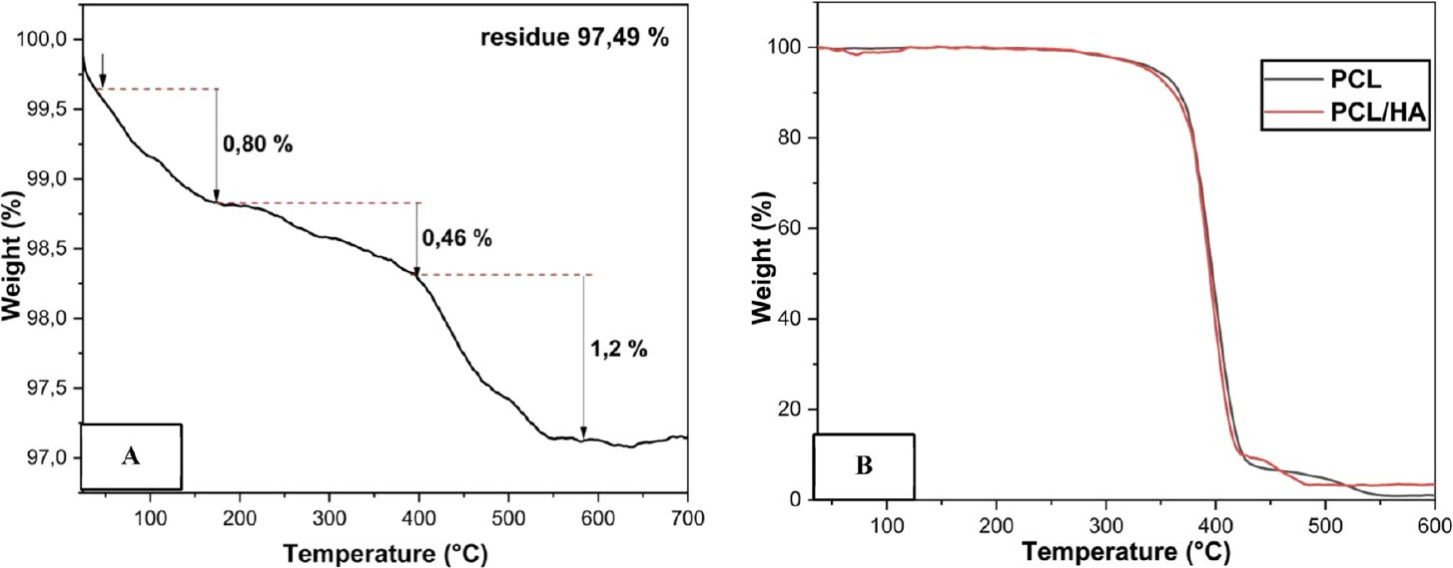
TGA curve of (A) hydroxyapatite and (B) for the scaffolds produced: PCL curve in black, PCL/HA curve in red.

The thermal properties of hydroxyapatite were evaluated by examining its mass loss through TGA. The outcomes of this analysis are presented in Figure 6A.

HA displays remarkable thermal stability, characterized by three discernible events with minimal mass loss. The initial event starts shortly after 40 °C and concludes around 190 °C, resulting in a mass decrease of 0.80%. This reduction is attributed to the desorption of physically adsorbed water from the material surface. The second event initiates around 190 °C and extends to approximately 375 °C, resulting in a 0.46% mass loss. It is associated with the removal of structural water, accompanied by an energy release. The third event, occurring between 375 and 600 °C, can be attributed to the reaction between two molecules of calcium monohydrogen phosphate, leading to the formation of pyrophosphate and water.^36^ Numerous studies have previously documented the thermally stable nature of hydroxyapatite. For instance, Szustakiewicz et al.^37^ reported a 3% mass loss for pure hydroxyapatite synthesized through coprecipitation in a temperature range up to 550 °C. Another example is the research conducted by Ghedjemis et al.,^38^ who observed three distinct mass loss events for hydroxyapatite derived from bovine and dromedary bones. This resulted in a total mass loss of 5.9% for HA obtained from dromedary bone and 5.8% from bovine bone in analyses conducted at temperatures up to 1500 °C. These findings corroborate that the material synthesized in this study exhibits properties consistent with those previously documented in the literature.

A comparative analysis was conducted between a scaffold produced without hydroxyapatite and one made with HA in terms of thermogravimetry, Figure 6B. The PCL scaffolds exhibited single-stage thermal degradation, occurring in the temperature range between 348 and 458 °C, with an approximate degradation percentage of 98.7% within the mentioned temperature range and a residual percentage of 1.3%. The mass loss in this temperature range can be attributed to the cleavage reaction of the chemical bonds in the polyester chains by pyrolysis.^39^ No significant mass losses were observed at temperatures below 340 °C, which could indicate the nonevaporation of the solvent used in the synthesis. Thus, it can be concluded that the solvent used in the electrospinning solutions evaporated during the electrospinning process. Scaffolds containing HA exhibited the same degradation stages as scaffolds without the ceramic content. However, in this case, they had a higher residual percentage (4.1%) due to the minimal decomposition of HA within the analyzed temperature range, as observed in this study. This behavior was also attended by Szustakiewicz,^37^ who, when producing porous poly(l-lactide)/hydroxyapatite scaffolds, observed that as the percentage of HA in the scaffolds increased, the rate of mass loss in thermogravimetric analysis decreased. This behavior is stable and standardized.

### 3.4 Swelling Rate Measurement

To assess the swelling behavior of electrospun scaffolds, the swelling test was conducted over 48 h. As specified, to analyze the degree of swelling, the dry weight of the samples was measured both before and after the liquid absorption process. The percentage of swelling exhibited by the studied scaffolds is shown in Table 1.

**Table 1:** Swelling Degree Values for Electrospun Scaffolds and Their Respective Standard Deviations (*n* = 3)

analysis time (h)	degree of swelling (%) PCL/HA
0,5	5.16 ± 0.035
1	10.7 ± 0.43
3	15.7 ± 3.8
6	18.1 ± 1.4
12	24.8 ± 0.24
24	36.5 ± 0.98
48	38.8 ± 0.080

Each value represents the average of three tests. Overall, the porosity exhibited by the structures (as observed through scanning electron microscopy) becomes an essential factor in facilitating swelling. It is noted that the swelling rate increased with the immersion time. Thus, there is a slight increase in absorption capacity after adding HA. This increase in hydrophilicity is associated with dipole interactions favored by the surface of HA, leading to water molecules being absorbed onto its surface.^40^ This liquid absorption capacity represents a crucial factor for tissue regeneration as it provides the necessary moisture to the target site to expedite the healing process. For a more comprehensive evaluation, an extended analysis is warranted.

### 3.5 Mechanical Analysis

The material’s mechanical properties are crucial for maintaining the scaffold structure during its deployment. Tissue rehabilitation after implantation directly relies on the material’s mechanical properties at both macro and microscopic scales. At a macro scale, the scaffold must be stable and robust enough for clinical manipulation, while at a microscale, cell growth, differentiation, and proliferation hinge upon the forces acting on the cells. Therefore, the scaffold must serve as adequate support to withstand applied forces and adequately transfer them to the encompassing cells and tissues.^41^

**Figure 7:**
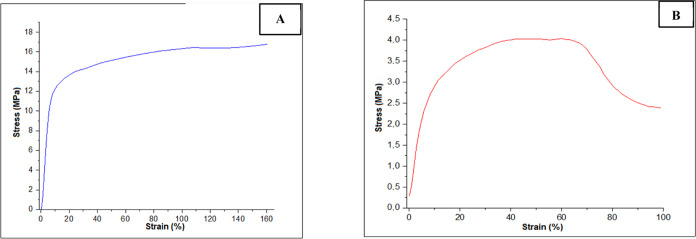
Typical stress–strain curves of scaffolds: (A) PCL; (B) PCL/HA.

A mechanical tensile test was conducted to examine stress versus strain to better comprehend the influence of hydroxyapatite nanoparticle insertion on the mechanical properties of electrospun scaffolds. The test yielded the following values: Young’s modulus, maximum stress, maximum elongation, and rupture stress. Figure 7 presents typical stress strain curves of PCL and PCL/HA scaffolds.

**Figure 8:**
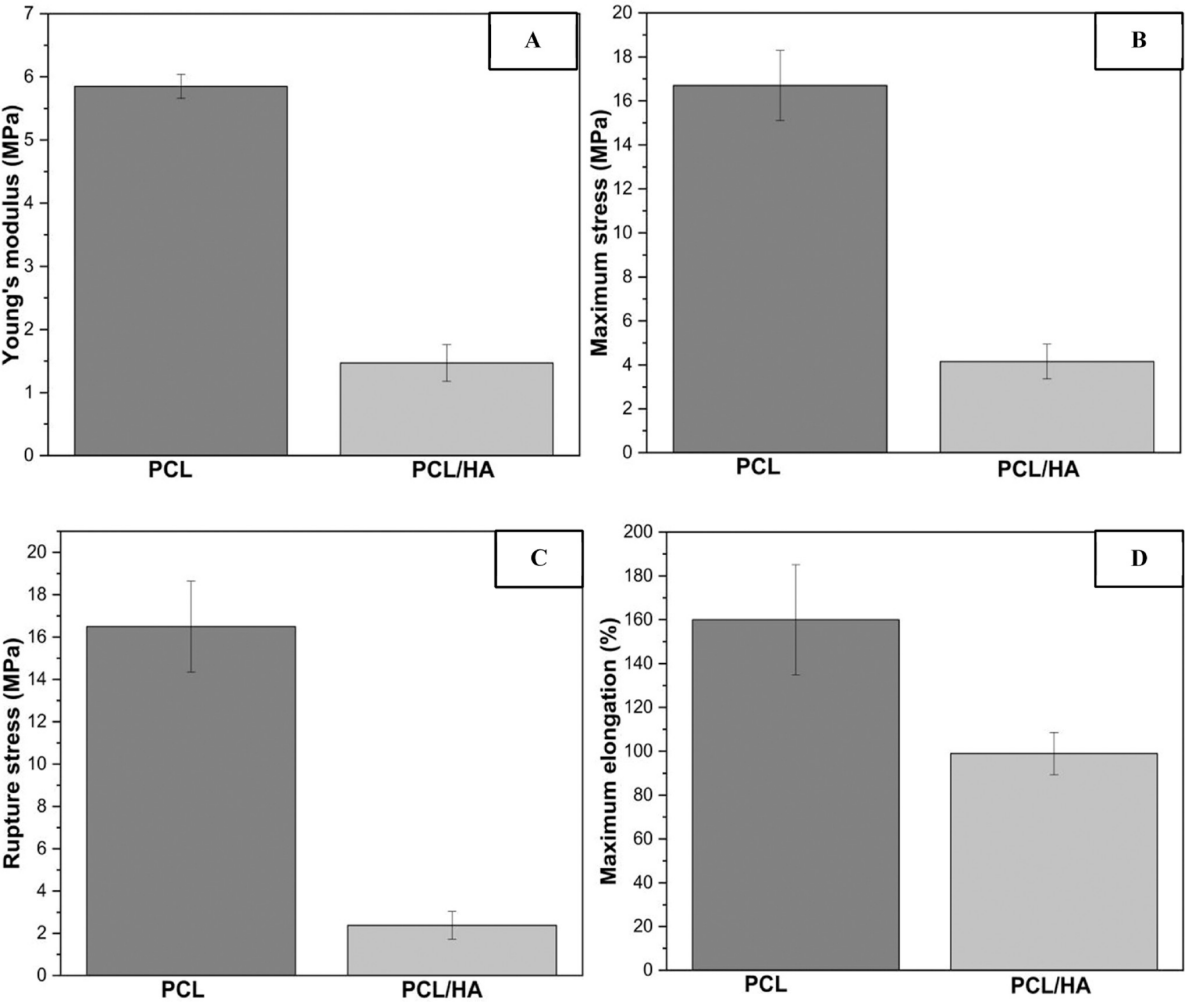
Values for the mechanical properties of the scaffolds and their respective standard deviations (*n* = 3): (A) Young’s modulus; (B) Maximum stress; (C) Rupture stress; (D) Maximum elongation.

As observed in Figure 7, the scaffolds undergo significant elongation with increasing stress. It is demonstrated that the tensile strength of the scaffolds decreases upon the insertion of hydroxyapatite nanoparticles. In other words, it can be affirmed that the insertion of HA reduces scaffold stiffness, as observed in the reduction of Young’s modulus values. PCL scaffolds without the ceramic content exhibited more significant plastic deformation. Conversely, PCL/HA films showed a lower plastic deformation value, thus experiencing a quicker interface rupture. The data obtained for each scaffold property are presented in Table 2 and Figure 8, respectively

**Table 2:** Values Obtained for Each Mechanical Property of Electrospun Scaffolds and Their Respective Standard Deviations (*n* = 3)

Scaffold	Young’s modulus	maximum stress	maximum elongation	rupture stress
PCL	5.85 ± 0.19	16.7 ± 1.6	160 ± 25	16.5 ± 2.1
PCL/HA	1.47 ± 0.29	4.16 ± 0.79	99 ± 9.6	2.38 ± 0.66

Therefore, a distinct mechanical behavior is observed for each type of scaffold produced. Several studies have confirmed that the incorporation of ceramic particles into polymeric matrices decreases mechanical strength.^41,42^ Kim et al.^43^ demonstrated that increasing the concentration of HA in a silk fiber polymeric matrix reduces the nanofibers’ mechanical strength. In this study, the authors obtained a lower maximum deformation value for scaffolds with a HA percentage above 20%. Similar results were also observed by Fu et al.,^42^ where the insertion of HA into PLGA nanofibers resulted in a lower percentage of plastic deformation. The absence of interfacial bonds between the ceramic content and the polymeric matrix can justify this decrease in the percentage of deformation. Thus, the HA particles functioned as a discontinuity factor between the polymeric chains, and the reduction in the distance between the particles, resulting from the increased percentage of hydroxyapatite in the scaffolds, substantially decreased toughness. This decrease in toughness results from a combination of defects produced around the HA nanoparticles caused by the concentration of tension under traction, resulting in microfractures. Therefore, an increase in the HA content in the fibers leads to an accelerated resistance breakdown during traction tests. Tensile tests demonstrated that the produced scaffolds exhibit good elasticity, allowing for extensions of ±160% in the absence of HA and ±99.0% with a 10% ceramic content. This makes the scaffolds potentially suitable for such applications. Regarding tensile strength, some authors have reported the possibility of bone regeneration with values lower than those obtained in this study. For instance, Ueyama et al.^44^ said the production of alginate and calcium chloride membranes applied to bone tissue engineering, favoring bone regeneration and exhibiting a resistance value of 0.017 MPa.

### 3.6 MTT Assay

**Figure 9:**
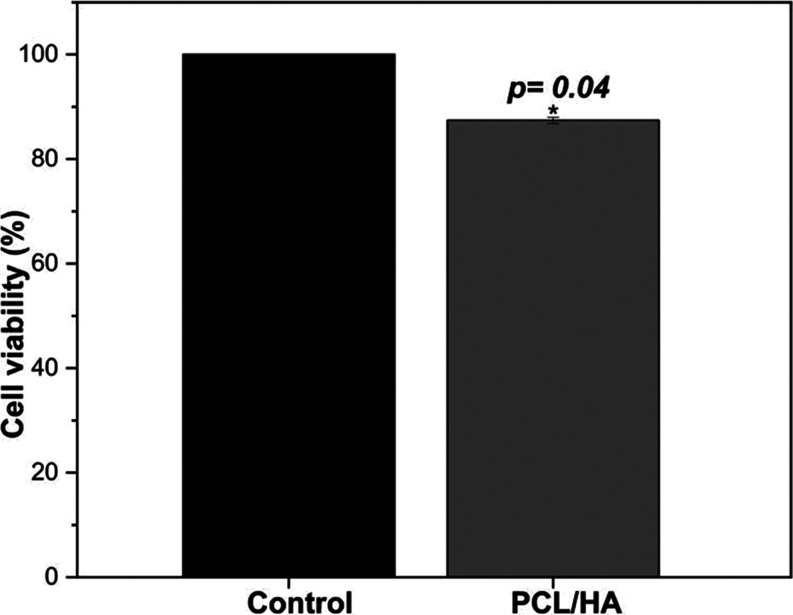
Cellular viability of Vero cells on PCL/HA electrospun scaffolds.

As observed in Figure 9, the produced scaffolds had a shallow negative impact, with cell viability exceeding the cytotoxicity threshold. According to ISO 10 993-5 standard, if cell viability is reduced to values below 70%, the material has cytotoxic potential. It is evident, therefore, that the produced biomaterial did not demonstrate toxicity. However, there was a slight reduction in cell viability in the scaffolds compared to the control, which can be attributed to the strong hydrophobicity of PCL, leading to cell adhesion difficulties. The samples with HA exhibited a viability percentage of 87%. In this regard, it was observed that incorporating nanohydroxyapatite into the polycaprolactone fibers influenced a slight increase in the material cytotoxic potential, possibly due to particle agglomerations along the fiber length.

### 3.7 Animal Study for Bone Regeneration In Vivo

No unfavorable occurrences were observed in the animals during the daily clinical examinations in the postoperative period. Both liquid and solid intake were reinstated on average 5 h after the surgical intervention, and no signs of discomfort or pain were reported. A slight edema was noticed in the surgical regions within the first 24 h. The wounds appeared clean without exudates, and the sutures remained intact, naturally falling off starting from the eighth day of the postoperative period.

### 3.8 Histopathological Analysis

**Figure 10:**
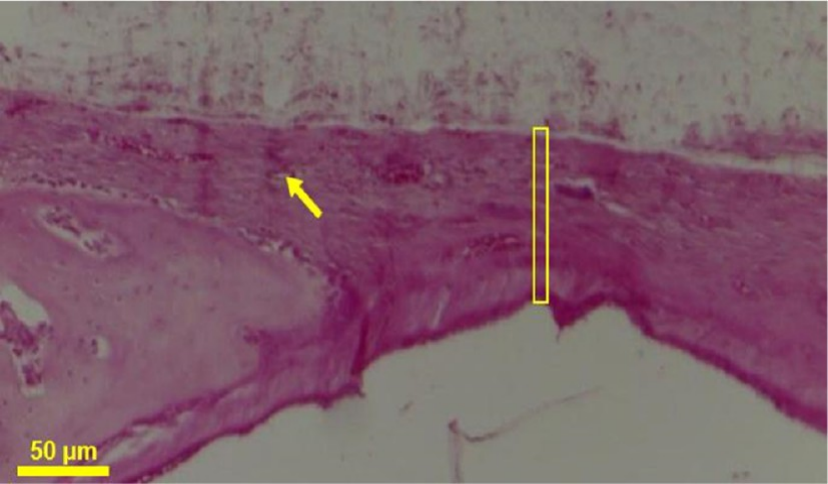
Photomicrograph in cross-section of the CG. Arrow—smallcaliber vessel; bar—connective tissue membrane. H.E. 10×.

After 30 days of the postsurgical period, histological analysis of the bone fragments in the groups treated with scaffolds (GE) and in the control group (GC) revealed the presence of a connective tissue membrane covering the surgical bone defect, extending to the adjacent periosteum of the affected area. In the experimental group (GE), there was an increase in the density of collagen fibers, which remained integrated with the bone margin, accompanied by considerable vascularization of small and medium-sized vessels. Intense osteogenic activity was noted along the interface between the scaffolds and the mature bone tissue at the margin of the surgical defect, evidenced by the abundant presence of osteoblasts and bone lacunae surrounded by osteoclasts (Figure 10).

**Figure 11:**
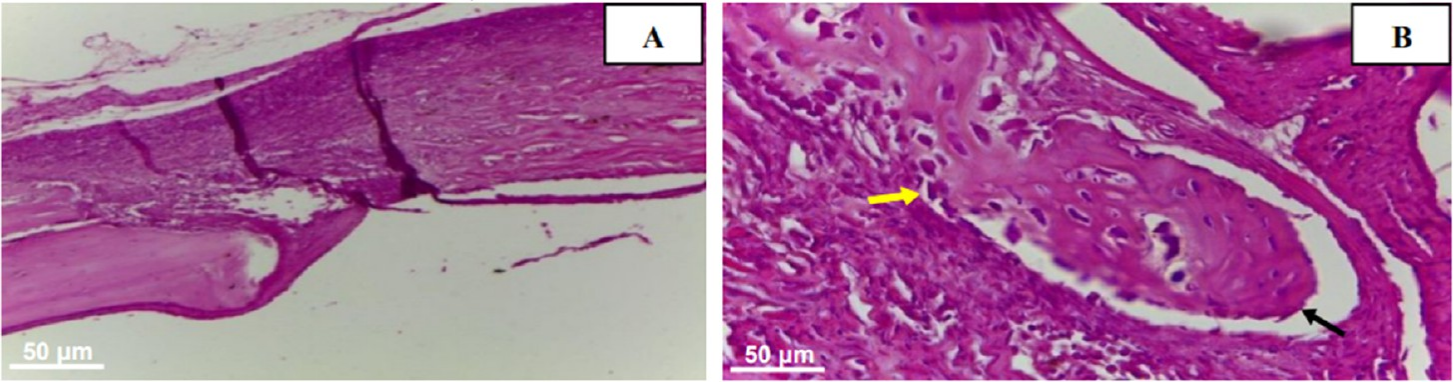
Photomicrograph in cross-section of the EG. In (A), evidence of matrix osseointegration at the bone edge is highlighted. In (B), the yellow arrow indicates osteoblast presence, while the black arrow emphasizes newly formed bone tissue. H.E. (A) 10× and (B) 40×.

Contrasting results were observed in the control group, where the membrane covering the bone defect resembles periosteal proliferation, appearing thinner and with limited vascularization, forming discrete granulation tissue (Figure 11).

In both samples, there is a precise observation of regenerative bone formation, either originating from the edges of the bone defect in the control group or at the margins and dispersed areas throughout the scaffold in the experimental group. This process involves a combination of fibrous connective tissue and osteoid, accompanied by a significant presence of osteoblasts around the newly formed bone region.

**Figure 12:**
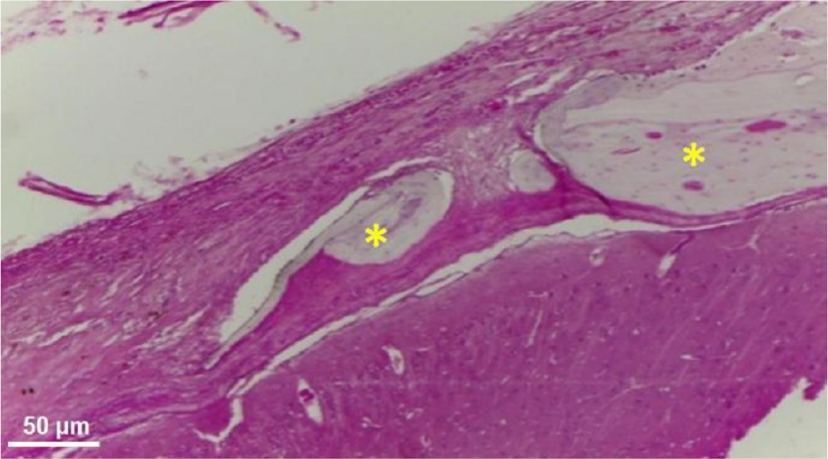
Cross-sectional photomicrograph of the EG at 30 days. Asterisk indicating newly formed bone tissue infiltrating the remnants of the scaffold. H.E. 10×.

After 30 days postsurgery, granulation tissue was observed in the control group (CG), along with regions of newly formed bone in both groups. In the CG, new bone formation appeared around the edges of the bone defect and in minor dispersed points within the connective tissue. In the experimental group (EG), a substantial amount of amorphous tissue resembling newly formed bone was infiltrating into the scaffold’s remaining areas (Figure 12).

**Figure 13:**
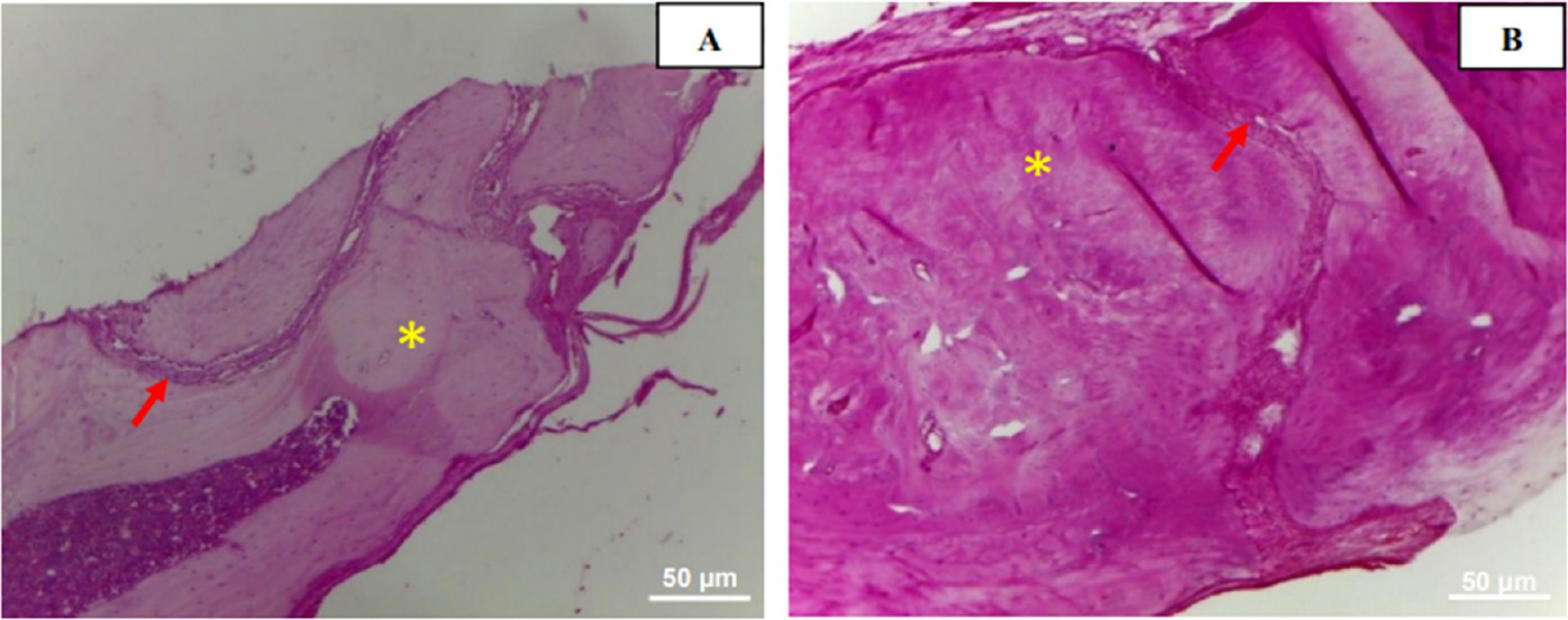
Cross-sectional photomicrograph of the EG. In image (A), yellow asterisk indicates osteoid presence and a red arrow pointing to a fibrous cord. In (B), an asterisk highlighting mature bone tissue and an arrow showing a fibrous cord. H.E. 10×.

In the experimental group (EG), fibrous connective tissue with a significant presence of osteoblasts was identified along the newly formed bone tissue. Additionally, the formation of bone trabeculae composed of mature bone and areas of osteoid was observed, interspersed with cords of connective tissue (Figure 13). These findings characterize the osteoconductive and osteoinductive capacity of the extracellular matrix derived from bovine sclera.

Based on the results of the macroscopic assessments in this study, it is evident that choosing rats as a model to investigate bone regeneration was crucial. This selection is attributed not only to the species’ resilience and excellent healing capacity but also to the ease of manipulation and favorable access to the surgical site, enabling the rapid and reproducible creation of bone defects. Furthermore, the low cost associated with conducting research using this animal model is noteworthy.

## 4 Conclusions

In this study, we addressed the preparation of functionalized fibrous scaffolds based on polycaprolactone (PCL) and hydroxyapatite for applications in bone tissue engineering. We demonstrated the development of a near-field electrospinning system adapted to a 3D printer, efficient in producing scaffolds with oriented fiber deposition. Near-field ES provides precise control over fiber deposition, reducing instabilities, thereby promoting the development of structurally robust systems for bone regeneration. This adapted technique emerges as a powerful tool for creating micro and nanometric diameter fibers and materials with controlled structure, particularly suitable for bone regeneration applications, with promising results.

## Supporting Information Available

Mechanical drawing of the bronze piston; mechanical drawing of the adapter bushing for syringe, adapter bushing for syringe; syringe holder to replace the print head; and EDS spectrum for PCL/HA scaffolds (PDF)
