# Supplementary material for: Development of Functionalized Poly(ε-caprolactone)/Hydroxyapatite Scaffolds via Electrospinning 3D for Enhanced Bone Regeneration
Source: ACS Omega. 2024 Oct 30;9(45):45035–46. doi: 10.1021/acsomega.4c05264 (PMC11561599; doi:10.1021/acsomega.4c05264)

Supporting Information

Development of Functionalized Poly(ε-caprolactone)/Hydroxyapatite Scaffolds via Electrospinning 3D for Enhanced Bone Regeneration

Maria José da Silva Lima[1], Etelino Feijó de Melo[2], Kleber Gonçalves Bezerra Alves[3], Fabrício Bezerra de Sá[4] and Severino Alves Júnior[1]*

[1]Departamento de Química Fundamental, Universidade Federal de Pernambuco, Recife, PE 50670-901, Brazil.

[2]Instituto Federal de Educação, Ciência e Tecnologia de Pernambuco, Vitória de Santo Antão, PE, 50740-540, Brazil

[3]Departamento de Engenharia Mecânica, Universidade Federal de Pernambuco, 50670-901 Recife, PE, Brazil.

[4]Departamento de Morfologia e Fisiologia Animal, Universidade Federal Rural de Pernambuco, Recife, PE 52171-900, Brazil.

Figure S1. Mechanical drawing of the bronze piston

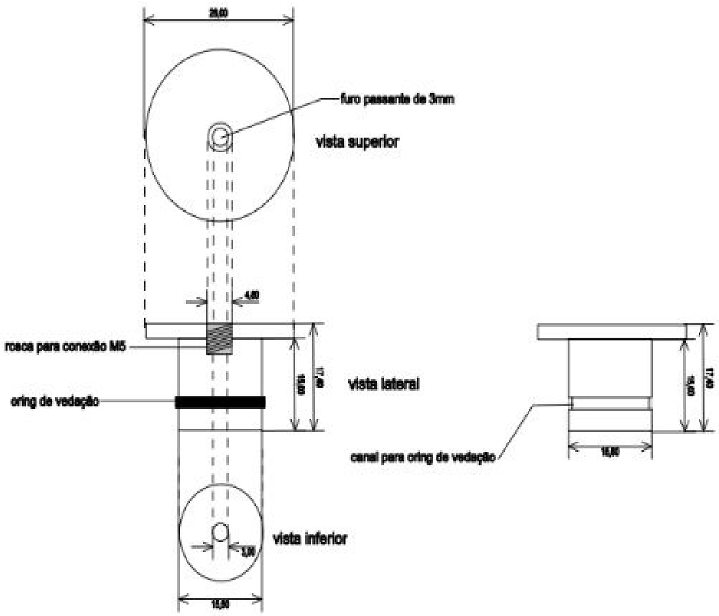

Figure S2. Mechanical drawing of the Adapter bushing for syringe

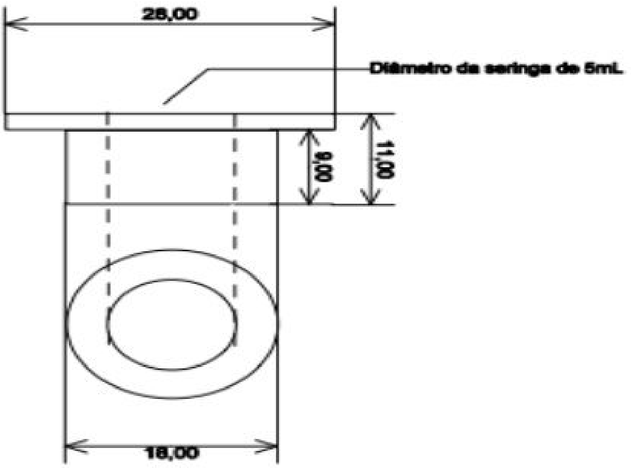

Figure S3. Adapter bushing for syringe

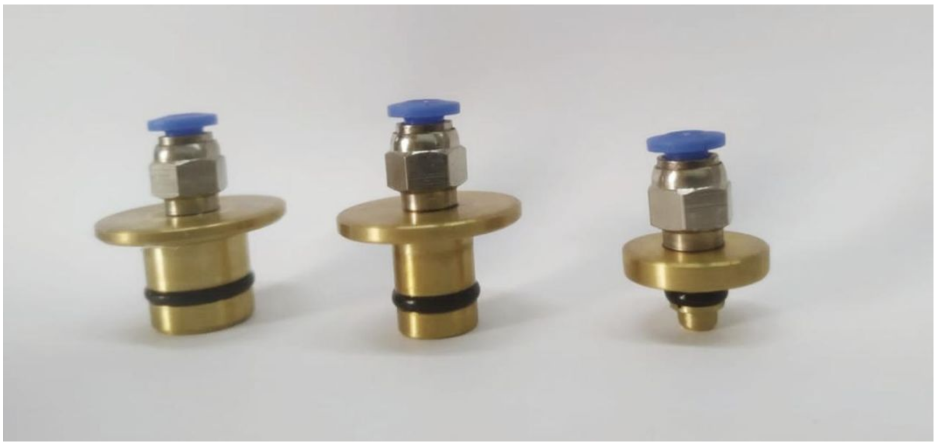

Figure S4. Syringe holder to replace the print head

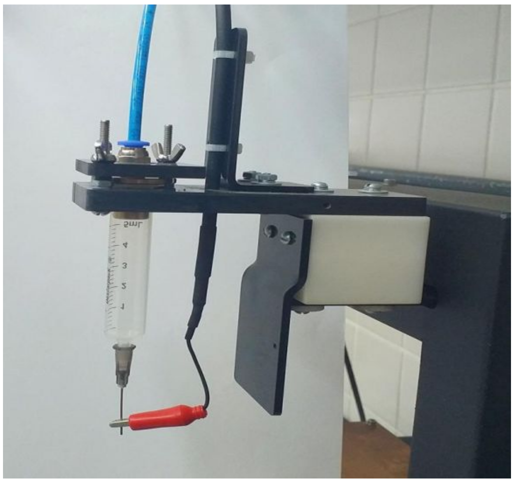

Figure S5. EDS map: (a) for the PCL/HA scaffold; (b) carbon map; (c) oxygen map; (d) calcium map; (e) phosphorus map,

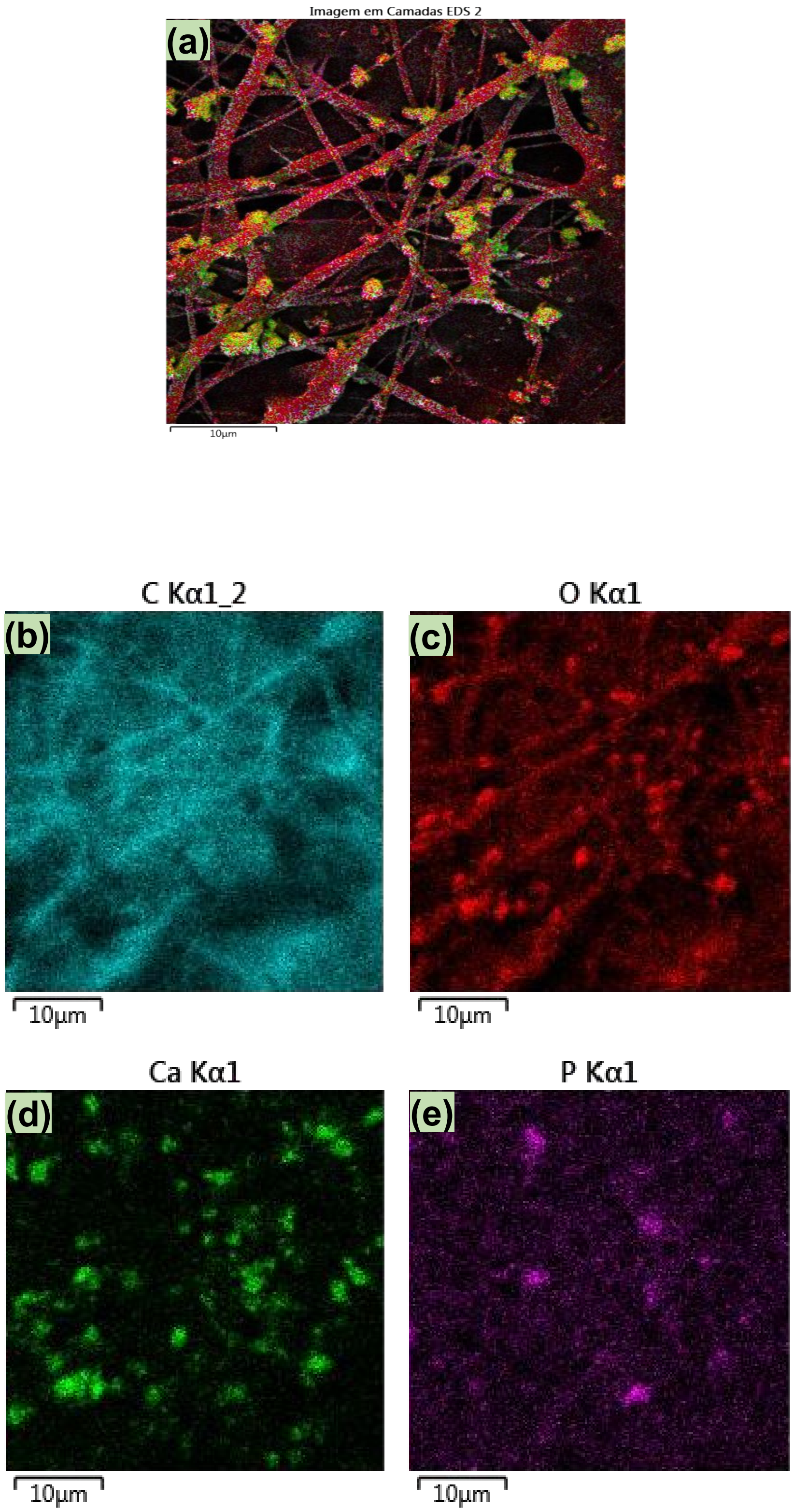

Figure S6. EDS spectrum for PCL/HA scaffolds

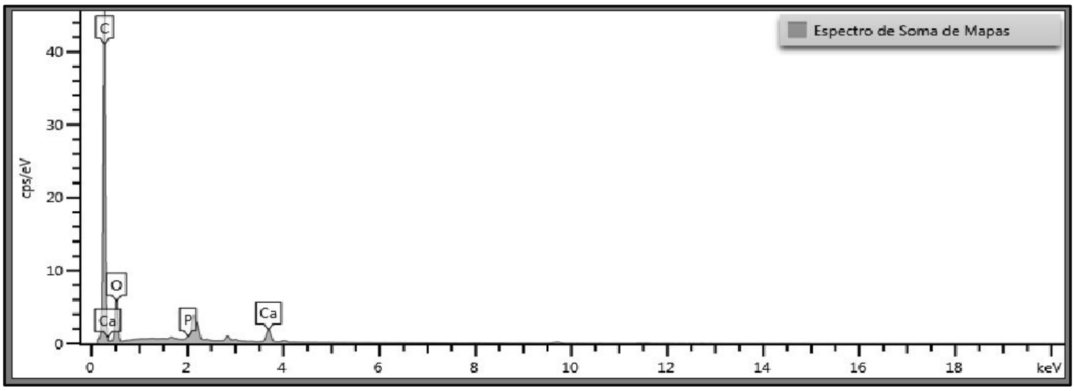

Figure S7. SEM images for oriented writing of fibers with a checkered profile: (a) 100 kx magnification; (b) 200 kx magnification

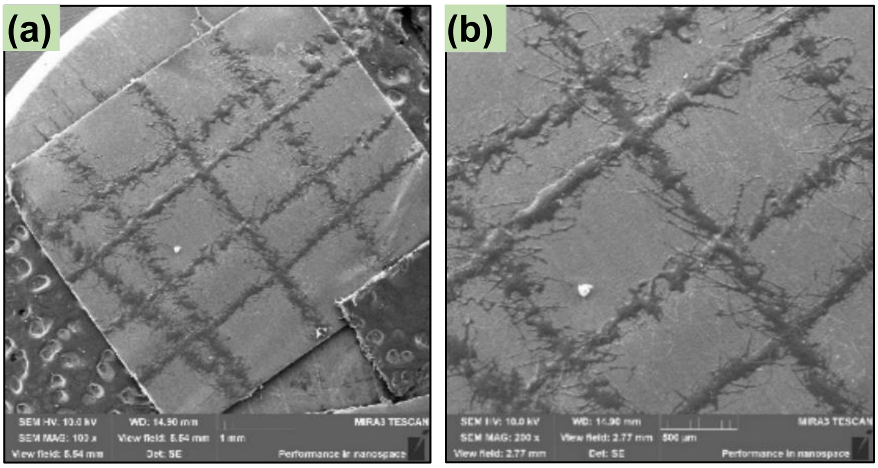

Figure S8. Optical microscopy images for scaffolds with oriented fiber writing

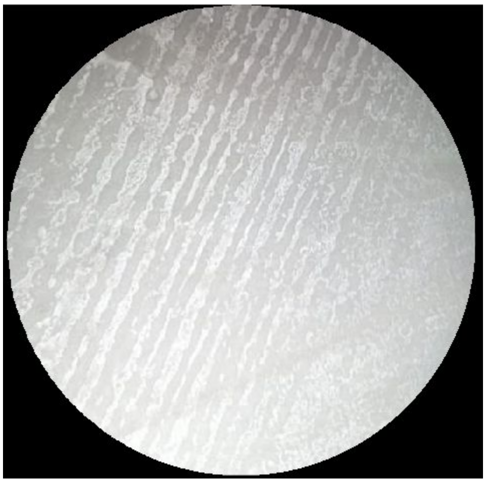

Supplement: Supplementary file 1 — ao4c05264_si_001.pdf [file ao4c05264_si_001.pdf]
